# Development of n‐type ZnAlInO Semiconductor Materials for Thermoelectric Generators in Aerospace Applications

**DOI:** 10.1002/open.202500082

**Published:** 2025-04-13

**Authors:** Enes Kilinc, Fatih Uysal, Mucahit Abdullah Sari, Huseyin Kurt, Erdal Celik

**Affiliations:** ^1^ Faculty of Engineering Department of Mechanical Engineering Karabuk University Demir‐Celik Campus 78050 Karabuk Turkiye; ^2^ Faculty of Technology Department of Mechanical Engineering Sakarya University of Applied Sciences Esentepe Campus 54187 Serdivan Sakarya Turkiye; ^3^ Faculty of Technology Department of Metallurgical and Materials Engineering Sakarya University of Applied Sciences Esentepe Campus 54187 Serdivan Sakarya Turkiye; ^4^ Faculty of Engineering Department of Mechanical Engineering Necmettin Erbakan University 42090 Meram Konya Turkiye; ^5^ Faculty of Mechanical Engineering Department of Mechanical Engineering Istanbul Technical University Gumussuyu 34437 Beyoglu Istanbul Turkiye; ^6^ R&D and Technology Directorate Turkish Aerospace Industries Inc. Central Campus 06980 Kahramankazan Ankara Turkiye; ^7^ Rectorate Bingol University 12000 Bingol Turkiye

**Keywords:** semiconductivity, sol–gel, thermoelectrics, ZnAlInO

## Abstract

Thermoelectric conversion is a system that can convert heat energy originating from temperature difference into electrical energy. Although it has many advantages in terms of usage, research is required to acquire high‐efficiency thermoelectric materials due to their low efficiency. Herein, Al‐ and In‐doped ZnO semiconductor thermoelectric material is synthesized, produced, and examined for use in the production of thermoelectric generators that can be used in aviation applications. The synthesis of ZnAlIn powders is carried out by the sol–gel method. The xerogel is dried at 200 °C for 10 h, and the dried material is then calcined at 600 °C for 4 h in an atmospheric oven to obtain ZnAlIn material. The obtained powder is then compressed with a cold press to produce pellet samples. Pellet samples are sintered in an atmospheric furnace at 1350 °C for 36 h and are made ready for measurements. Comprehensive characterization and analysis of microstructural and structural properties are performed by Fourier transform infrared spectroscopy, differential thermal analysis‐thermogravimetry, X‐ray photoelectron spectroscopy, scanning electron microscopy, and X‐ray diffraction methods. Seebeck coefficient and thermal capacity measurements are performed to determine thermoelectric properties. The results obtained from the study show that ceramic‐based ZnAlIn semiconductor thermoelectric material has the required efficiency for thermoelectric generator production.

## Graphical abstract

Herein, the synthesis and production of n‐type ZnAlInO semiconductor materials is achieved utilizing sol–gel and cold pressing methods for fabrication in aerospace thermoelectric generators applications. The Seebeck coefficient and power factor values increase with the addition of template samples, reaching a maximum of −162.63 μV K^−1^ and 0.44 mW mK^−2^, respectively, at 800 °C for Zn_0.96_Al_0.02_In_0.02_O.

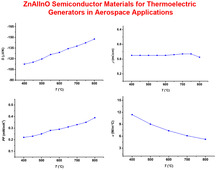

## 1 Introduction

Thermoelectric materials are an important alternative for aviation applications owing to their capacity to transform temperature differences into electricity energy and are an important research topic for electricity generation and thermal system administration in aerospace exploration and aviation.^[^ ^1^ ^]^ In spacecraft, thermoelectric generators (TEGs) could be a dependable substitute for solar panels to generate electrical power using radioactive decay or waste heat on long‐duration goals to distant celestial bodies.^[^ ^2^, ^3^ ^]^ These materials also facilitate precise temperature regulation, ensuring equipment longevity and performance.^[^ ^4^ ^]^ In aviation, thermoelectric materials can enhance energy efficiency and reduce environmental impact by recovering waste heat from engines and improving cabin climate control.^[^ ^5^ ^]^ Despite their potential, they are not currently widely used in aviation applications due to obstacles such as low efficiency, weight, and high costs.^[^ ^6^ ^]^

Research is centered on creating cutting‐edge materials with enhanced thermoelectric qualities, optimizing device designs for high performance, and investigating innovative manufacturing techniques for lower manufacturing costs.^[^ ^7^, ^8^ ^]^ The sol–gel technique is extensively employed in the synthesis of p‐type and n‐type ceramic semiconductors used in the production of TEGs. Calcium cobalt oxide (Ca_3_Co_4_O_9_), which is the basic component of one of the p‐type materials, was doped with silver (Ag) and rare earth elements such as lanthanum (La), ytterbium (Y) europium (Eu), and lutetium (Lu).^[^ ^9^, ^10^ ^]^ To synthesize, zinc oxide (ZnO) was doped with aluminum (Al) and indium (In) to form zinc aluminum indium oxide (ZnAlInO) powders.^[^ ^11^ ^]^

In further detail, the synthesis of ZnAlInO powders via Al and In doping of ZnO is an important development in the field of thermoelectric materials. In the literature, S. Sugihara et al.^[^ ^12^ ^]^ explored metal oxides, including ZnAlO, focusing on their thermoelectric properties and electronic structures. They found that doping ZnO with Al reduces electrical resistivity and enhances its semiconducting state. A. Zankat et al.^[^ ^13^ ^]^ analyzed the electrical characteristics of composites made with ZnO. Synthesizing ZnO nanoparticles via sol–gel and incorporating them into a Zn_0.95_Al_0.05_O matrix. They analyzed how varying ZnO nanoparticle content (0.5, 10, 15, and 20 wt%) affects the composite's resistance and reactance. T. Samerjai et al.^[^ ^14^ ^]^ examined flame‐spray‐made ZnInO nanoparticles, finding that the alloyed nanoparticles had smaller particle sizes and lower crystallinity compared to pure ZnO and indium oxide (In_2_O_3_). I. Haq et al.^[^ ^15^ ^]^ explored the impact of sintering temperature on the thermoelectric characteristics of ZnInO thin films produced via physical vapor deposition. Their study highlighted the growth of these films using thermal evaporation, targeting applications in thermoelectric power generation. Analysis of the Seebeck coefficient data revealed values ranging from 140.9 to 182.2 μV °C^−1^. Based on these findings, the study suggests that ZnInO thin films hold promise for future applications in thermoelectric power generation. Although there is extensive literature on metal oxide‐based thermoelectric materials for waste heat recycling with TEGs in automotive and aircraft implementation, there is a significant lack of ZnAlInO‐based thermoelectric materials. Except for our pioneering research,^[^ ^11^ ^]^ no research has investigated the energy efficiency of ZnAlInO‐based thermoelectric materials through the recovery of waste heat in aircraft or automotive exhaust systems. Our research aims to address this gap and establish the viability of ZnAlInO TEGs for these sophisticated applications.

Current research on ZnAlInO powders highlights significant advances in their characterization, synthesis, and use for thermoelectric implementation. Efforts in materials design, manufacturing methods, and performance improvement are driving innovation in conversion and energy harvesting, particularly in aviation. This study comprehensively investigates the properties of the solution prior to ZnAlInO powder synthesis utilizing the sol–gel technique, addressing gaps within the literature. Along with thermal, structural, and morphological investigations, it also includes turbidity measurements to evaluate precursor dissolution and analysis of the acidity of a solution using a pH meter. Sol–gel process optimization and thermoelectric property evaluation like Seebeck coefficient, power factor, thermal conductivity, and electrical resistivity, methods such as differential thermal analysis‐thermogravimetry (DTA‐TG), Fourier transform infrared spectroscopy (FTIR), X‐ray photoelectron spectroscopy (XPS), X‐ray diffraction (XRD), scanning electron microscopy (SEM), and thermoelectric measurements were used.

## 2 Experimental Section

### 2.1 Materials

In order to produce Zn_0.96_Al_0.02_In_0.02_O materials consisting of different stoichiometric ratios, production was carried out by doping Al and In elements into ZnO semiconductor material at different ratios. For this purpose, zinc nitrate, aluminum nitrate, and indium (III) nitrate hydrate precursor materials were used. These metal nitrates were dissolved in pure water, and glacial acetic acid was used as catalysts and chelate formers to accelerate the gelation process. The formulas and purity ratios of the materials used are given in **Table** **1**.

**Table 1:** Materials used in the production of n‐type semiconductors and their properties.

Chemical type	Formula	Precursors	Molar weight [g]	Purity [%]	Molar weight [g]
Precursor materials	Al(NO_3_)_3_.9H_2_O	Aluminum nitrate	375.13	98.00	375.13
	Zn(NO_3_)_2_.6H_2_O	Zinc nitrate	297.47	99.00	297.47
	In(NO_3_)_3_.xH_2_O	Indium (III) nitrate hydrate	300.83	99.999	300.83
Solvent	H_2_O	Distilled water	18.02	99.50	18.02
Chelating agent	CH_3_CO_2_H	Glacial acetic acid	60.05	99.85	60.05

### 2.2 Fabrication Process

**Figure 1:**
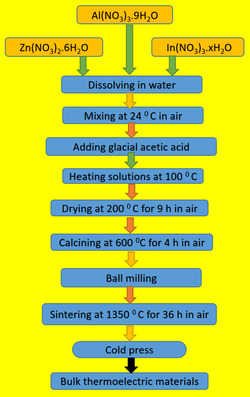
Diagrammatic representation of the production of solutions, powders, and bulk materials.

In the sol–gel technique used for thermoelectric materials, the production of the precursor solution is essential. The detailed flow chart in **Figure** **1** provides a comprehensive overview of the successive steps involved in that synthesis, giving a more thorough explanation of the procedure. The rationale for selecting the specific ratio of Al and In in Zn_0.96_Al_0.02_In_0.02_O is driven by a strategy aimed at enhancing thermoelectric properties. Doping ZnO with Al and In was performed to optimize the material's electrical conductivity, Seebeck coefficient, and power factor. This ratio was determined with careful consideration of the distinct properties of both elements and their effects on the ZnO matrix. Al generally enhances n‐type semiconductor behavior, while the influence of In helps to reduce thermal conductivity, thereby improving thermoelectric performance. Therefore, the stoichiometric chemistry was employed to determine the optimal composition. This composition selection may have resulted from an experimental optimization process aimed at achieving better thermoelectric performance and lower thermal conductivity. With this ratio, it is likely that a balance between the desired electrical and thermal properties was successfully achieved.

The formulation of the precursor solution for ZnAlInO material followed a methodical approach, which included dissolving nitrates corresponding to component elements (Zn, Al, and In) in pure water, maintaining predefined cationic ratios. Glacial acetic acid was added to the solution to accelerate dissolution and then stirred on a magnetic stirrer at 120 °C to increase uniformity. Glacial acetic acid, acting as a chelating agent, facilitated the formation of xerogels. These procedures were reiterated for all components in the indicated stoichiometric ratios to produce semiconductor materials.

Following the successful synthesis of xerogels in a homogeneous structure by the sol–gel method, sintering and heat treatments were applied in an open‐atmosphere electric furnace. After 10 h of drying at 200 °C to eliminate humidity and gases, the ZnAlInO powders were calcined for 4 h at 600 °C as the last stage. Then, the powder was pressed into shaped pellets by cold pressing (CP) under 1100 MPa pressure. The pellet samples were sintered at 1350 °C for 36 h to form bulk samples containing Zn_0.96_Al_0.02_In_0.02_O composition.

### 2.3 Characterizations

Prior to manufacture, the turbidity and pH levels of the produced solutions were examined since they have an impact on the structural characteristics of thermoelectric materials. Turbidity was measured using a VELP TB1 Model turbidity meter with standard solutions for coating implementation in a container with a height of 50 mm and a diameter of Ø25 mm. Turbidity calibration was performed with Formazan in the range of 0–1000 ntu. A conventional tabletop pH meter fitted with a Hanna HI‐1230 electrode was then used to measure the pH values of optically clear liquids. The obtained results were used to evaluate the alkaline or acidic nature of the solutions.

Optimizing the thermal treatment of the materials that are synthesized, the Perkin Elmer STA 6000 model differential thermal analysis‐thermogravimetry (DTA‐TG) device was utilized to monitor the reactions at various temperatures. 8 mg samples taken from gels desiccated at 200 °C were examined. The specimens were heated gradually to set temperatures in the device, and weight losses and reaction types in ZnAlInO materials were observed at gradual temperature increases from ambient temperature to 1000 °C. This application allowed the determination of suitable heat treatment conditions for the materials.

Fourier transform infrared spectroscopy (FTIR) analysis was conducted to examine organic structures within the xerogels synthesized through sol–gel processing and powders obtained from thermal processes. This round of the investigation was conducted using a ThermoScientific NICOLET iS10 FTIR analyzer. In order to examine the n‐type ceramic characteristics of ZnAlInO, samples were heated between 200 and 600 °C and then submitted to FTIR analysis.

Using a ThermoScientific ARL X'tra brand diffractometer, XRD analysis was used to describe the component phases in the produced ceramic semiconductor powder materials. Cu K_α_ radiation (λ = 0.15405 nm) in the Cu tube with a current of 20 mA and a voltage of 30 kV was used for the tests.

Thermo Scientific K‐Alpha X‐ray photoelectron spectroscopy (XPS) was utilized to investigate the surface chemical characteristics of ZnAlInO materials. The photoelectron emission from an Al K_α_ X‐ray source (1486.6 eV) at 150 W was examined using a hemispherical analyzer in fixed retardation ratio mode (pass energy: 11.75 eV, resolution: <0.51 eV) at a take‐off angle (α) ranging from 30° to 70°.

The morphology of ZnAlInO materials was characterized by utilizing SEM with a Zeiss Ultra Plus Gemini system at 20 kV, providing detailed perceptions of the surface microstructure and nanoscale morphology.

Measurement of thermoelectric properties such as electrical conductivity and Seebeck coefficient of bulk samples was quantified at increments of 50 between 400 and 800 °C with a Linseis LSR‐3 system. Resistivity was measured by passing a 100 mA current through the sample using a four‐point probe method. Thermal conductivity (κ) was obtained from κ = αρ*c*_p_, where α represents thermal diffusivity (measured by Netzsch LFA 457 MicroFlash Apparatus), the density (ρ), and the specific heat capacity (*c*_p_). *C*_p_, assessed using the Dulong–Petit law,^[^ ^16^ ^]^ was calculated as 0.618 J gK^−1^, and sample densities, determined by Archimedes’ principle, were 4.15 g cm^−3^. The thermoelectric properties were determined over the given temperature range by repeating measurements on at least three samples and calculating the arithmetic mean.

## 3 Results and Discussion

### 3.1 Solution Characteristics

The production of Zn_0.96_Al_0.02_In_0.02_O materials was carried out by using transparent solutions obtained from solvents, chemical precursors, and chelating substances. These solutions are made by dissolving substances that contain metal cations, including Zn(NO_3_)_2_.6H_2_O, Al(NO_3_)_3_.9H_2_O and In(NO_3_)_3_.*x*H_2_O with the polar aqueous solvent distilled water. Glacial acetic acid (CH_3_CO_2_H) plays multiple roles as a pH modulator, acid catalyst in sol–gel chemistry, stabilizer, solvent, and chelating agent. The bidentate structure affects the characteristics of the materials synthesized by sol–gel processes and enables them to bind to metal atoms.^[^ ^17^, ^18^ ^]^

Turbidity measures the optical clarity of liquids, like distilled water, by quantifying light scattering due to solution constituents upon exposure to a light beam.^[^ ^19^ ^]^ Higher turbidity indicates increased light scattering intensity, reflecting the efficiency of dissolving powdered precursors such as Zn(NO_3_)_2_.6H_2_O, Al(NO_3_)_3_.9H_2_O and In(NO_3_)_3_.*x*H_2_O in solutions, assessed through nephelometric turbidity unit (ntu) readings spanning 0 to 1000. The “minimum turbidity value” indicates the efficacy of precursor dissolving, essential for attaining the correct stoichiometry through full dissolution and suitable sintering procedures.^[^ ^20^, ^21^ ^]^ Turbidity values approaching 0 ntu or within the range of 0–50 ntu signify a uniform synthesis of transparent precursors, crucial for the fabrication of stoichiometric Zn_0.96_Al_0.02_In_0.02_O structure without necessitating further characterisation procedures. Turbidity test results of 0.52 ntu (**Table** **2**) highlight successful precursor dissolution and clear solution formation, critical for subsequent processes. Undissolved precursors lead to non‐homogeneous ceramics exhibiting stoichiometric deficiencies.^[^ ^22^, ^23^ ^]^ Minimum turbidity values confirm successful manufacturing of desired material compositions, aligned with XRD and XPS analyses.

**Table 2:** Turbidity and pH measurements of formulated solutions.

Solutions	Turbidity value [ntu]	pH value
Zn_0.96_Al_0.02_In_0.02_O	0.52	1.26

Sol–gel precursors exposed to reactions in solvent (H_2_O) and chelating agent (CH_3_CO_2_H). The partial load model predicts these reactions effectively, especially in non‐neutral solutions (pH ≠ 7), where Zn(NO_3_)_2_.6H_2_O, Al(NO_3_)_3_.9H_2_O and In(NO_3_)_3_.*x*H_2_O precursors, alongside water molecules, carry partial charges. pH values for prepared solutions are 1.26, indicating acidity (see Table 2). The pH plays a crucial role in gel formation, affecting structure‐acidic conditions from branched structures, while fundamental conditions yield clusters. The pH also influences condensation, complexation, and hydrolysis, critical in achieving transparent solutions. Sol–gel technique is vital for complex oxide synthesis (e.g., Zn_0.96_Al_0.02_In_0.02_O), leveraging salt precursors, chelating agents, and solvents. Assessing solution acidity is essential before powder production commences.

Hydrolysis refers to the removal of protons from a solvated metal cation (*M*), in which one or more surrounding water molecules in the primary solvation shell lose a proton. As a result, the aquo ligand molecule H_2_O attached to the metal is converted to a hydroxo ligand, OH^−^, if only one proton is released, or to an oxo ligand, O^−2^, if two protons are removed.^[^ ^18^, ^19^, ^24^ ^]^ In research where salt‐based precursors were dissolved in water and toluene, complexation occurred during the reactions within these solvents. As a result, Zn, Al, and In undergo extensive condensation and precipitate as hydroxides and oxo‐hydroxides with the formulas Zn(OH)_2_, Al(OH)_3_, and In(OH)_3_, respectively. After hydrolysis, condensation reactions lead to the formation of polynuclear complexes that incorporate two metal atoms. As previously mentioned, the chelating ligand, glacial acetic acid (CH_3_CO_2_H), plays a crucial role in these condensation processes. Condensation, particularly in aqueous solutions, arises from either olation or oxolation reactions. In either case, careful regulation is essential, as oxygen can significantly speed up the reaction, sometimes requiring an argon atmosphere for stabilization. In the olation process, hydrogen transfers to an OR ligand, as outlined in Reactions (1–3).

(1) $$\text{Zn}{\left({\text{NO}}_{3}\right)}_{2}+2H_{2}O\to \text{Zn}{\left(\text{OH}\right)}_{2}+2{\text{HNO}}_{3}$$

(2) $$\text{Al}{\left({\text{NO}}_{3}\right)}_{3}+3H_{2}O\to \text{Al}{\left(\text{OH}\right)}_{3}+3{\text{HNO}}_{3}$$

(3) $$\text{In}{\left({\text{NO}}_{3}\right)}_{3}+3H_{2}O\to \text{In}{\left(\text{OH}\right)}_{3}+3{\text{HNO}}_{3}$$

In the second case, in oxolation, hydrogen transfers to an OH group, following Reactions (4–6).

(4) $$2\text{Zn}{\left(\text{OH}\right)}_{2}\;\to {\text{ Zn}}_{2}{\left(\text{OH}\right)}_{2}O_{4}\;+{\text{ H}}_{2}O$$

(5) $$2\text{Al}{\left(\text{OH}\right)}_{3}\;\to {\text{ Al}}_{2}O_{3}\;+\;3H_{2}O$$

(6) $$2\text{In}{\left(\text{OH}\right)}_{3}\;\to {\text{ In}}_{2}O_{3}\;+\;3H_{2}O$$

Hydrolysis and condensation continue simultaneously, gradually forming a three‐dimensional network that eventually leads to a solid phase. That procedure is expedited by heat, as the rates of both reactions increase with temperature. The kinetics of hydrolysis and condensation, and therefore the total process and kinds of polymers, are influenced by pH, allowing for the synthesis of a diverse array of materials with differing structures. These structures include linear polymers, particles that are dense and colloidal, and smaller, cross‐linked polymer clusters with weaker bonding.^[^ ^19^ ^]^

Van der Waals forces and the creation of covalent or noncovalent bonds are two of the factors that control gelation, the process by which particles or molecules group together in a liquid media.^[^ ^19^ ^]^ It is significant because nitrate (NO^3−^) ions have a tendency to promote the production of tiny polymers with a more crystalline structure. It is clear that anions have a major influence on the structural characteristics of sol–gel materials as these polymers are essential building blocks in the creation of the final solid substance. Nonetheless, these systems may present some difficulties due to the existence of various anions. In particular, the homogeneity attained during the sol–gel processing phases may be disturbed by nitrate ions, which are prone to crystallize in the course of the drying step.^[^ ^18^, ^19^, ^24^ ^]^ In powder production, such issues are mitigated, as nitrate crystallization may actually help reduce particle size. This effect is crucial because manipulating particle size significantly impacts microstructural properties and, consequently, the thermoelectric characteristics of the resultant powders. Therefore, carefully managing these dynamics is essential for optimizing sol–gel methods for a variety of implementations.

### 3.2 Thermal Analysis

In the stoichiometric synthesis of thermoelectric ceramics, the pyrolysis stage is crucial. It involves careful heating of Zn_0.96_Al_0.02_In_0.02_O precursor solution to prevent negative impacts on the ceramic's stoichiometry and thermoelectric properties. Water, organic complexes, chelating agents, and chemical changes all burn during pyrolysis, which can have an impact on the quality of the finished product. To mitigate these effects, precursor solutions are desiccated at 200 °C for 10 h in the air before heat treatment. DTA‐TG analyses under controlled air conditions are used to monitor thermal transitions and chemical processes, aiming to optimize the synthesis and improve the ceramic's structural soundness and thermoelectric performance.

**Figure 2:**
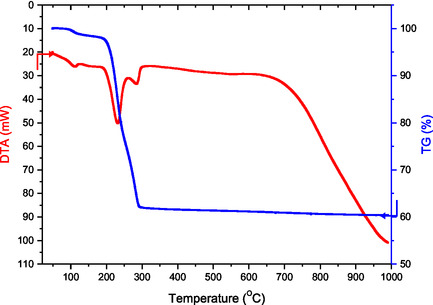
The DTA‐TG curves for Zn_0.96_Al_0.02_In_0.02_O materials dried at 200 °C for 10 h in air.

**Figure** **2** presents the DTA‐TG profiles of Zn_0.96_Al_0.02_In_0.02_O powder specimens after being desiccated at 200 °C for 10 h in atmospheric air. **Table** **3** describes the reactions and formation temperatures in detail. Thorough DTA‐TG analyses reveal a distinct endothermic reaction between 95–105 °C, accompanied by a weight loss of about 3%, ascribed to the evaporation of remaining moisture within the gel matrix.^[^ ^25^ ^]^ Another significant endothermic transition occurs between 200 and 320 °C, indicating the combustion of organic materials and carbonaceous substances, evidenced by a corresponding mass loss. Gas release, including H_2_O, CO, CO_2_, and NO_*x*_, is noted between 100 and 300 °C, further explaining the observed weight loss. A secondary endothermic peak appears between 200 and 250 °C in the DTA curve, with an associated weight loss of around 37%, linked to the combustion of organic elements in the gel matrix. The oxidation of material elements and the beginning of phase formation of ZnO, Al_2_O_3_ and In_2_O_3_ are responsible for the most noticeable endothermic peak, which is seen between 300 and 350 °C and corresponds to a weight loss of almost 40%. Notably, thermal treatment at moderate temperatures, such as 500 °C, reduces the anion‐to‐cation ratio to 0.05, producing a highly pure structure, although with nitrates, this can be a potentially explosive process.^[^ ^18^, ^19^ ^]^ Furthermore, a reaction at about 700 °C signifies that the components are beginning to form a secondary phase. According to these findings, the ideal thermal processing temperature for ceramic materials based on n‐type ZnAlInO is 600 °C. Another endothermic transition at around 670 °C indicates the oxidation of Zn, Al, and In components. While some powder materials show a single reaction between 300 and 500 °C, multiple reactions are observed due to the combustion of organic compounds, oxidation, and phase formation. The decomposition of nitrates also contributes to weight loss. Topotactic reactions between 500 and 800 °C lead to non‐stoichiometric structures. At 700 °C, a weight reduction and low‐energy endothermic event suggest a transition to a secondary phase.^[^ ^26^ ^]^ Thus, 600 °C is shown to be the ideal thermal processing temperature for n‐type ZnAlInO‐based materials.

**Table 3:** Heat treatment schedules for ZnAlInO ceramic material.

Reaction type	Properties/Process	Optimum temperature values [°C]	Reaction temperature range [°C]	Duration time [min]
Endothermic	Drying	200	25–200	120
Endothermic	Combustion of organics	300	200–250	30
Endothermic	Phase transformation	600	300–350	240

The heat treatment process for Zn_0.96_Al_0.02_In_0.02_O ceramic material was designed as follows, guided by DTA‐TG analysis: a) an initial drying phase at 100 °C for 3 h to ensure the removal of residual solvents; b) gradual decomposition of organic binders and nitrates at 200 °C for 10 h; c) calcination to promote phase formation and oxidation at 600 °C for 4 h; and d) final densification and grain growth through sintering at 1350 °C for 36 h in an oxidizing atmosphere. The intended material qualities and the ceramic phase's structural integrity are guaranteed by these procedures.

### 3.3 FTIR Analysis

**Figure 3:**
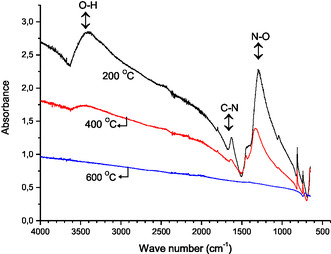
The FTIR analysis results of Zn_0.96_Al_0.02_In_0.02_O powder materials heat treated at 200, 400, and 600 °C.

An experimental protocol was implemented to prepare ceramic samples for FTIR spectroscopic analysis, targeting ZnAlInO materials. The specimens underwent sequential heating cycles at 200, 400, and 600 °C, then by detailed characterization using FTIR spectroscopy. **Figure** **3** illustrates the resulting FTIR spectra of the n‐type semiconductor ceramic powders, revealing prominent absorption bands associated with oxygen–hydrogen (O—H), nitrogen–oxygen (N—O), carbon–nitrogen (C—N), and metal–oxygen (M—O) bonds.

The sol–gel synthesis method, utilizing water as the solvent, naturally results in the presence of O—H bonds within the material, as reported in prior studies.^[^ ^27^ ^]^ These bonds are identified by distinct peaks in the 3250–3600 cm^−^^1^ region, attributed to O—H stretching vibrations. Thermal treatments at 200, 400, and 600 °C progressively reduce the intensity of these peaks, indicating the gradual removal of adsorbed water molecules. Notably, the O—H stretching peak is prominent at 200 °C, weakens significantly at 400 °C, and disappears entirely at 600 °C.

In the analysis of FTIR spectra of samples subjected to varying thermal processes, distinct absorption bands are observed at specific wavenumbers: 1384, 1075, and 825 cm^−^^1^. The absorption band at 1384 cm^−^^1^ corresponds to the vibrational modes of NO_3_^−^ groups, while those at 1075 and 825 cm^−^^1^ are associated with carboxylic groups’ vibrational characteristics. The utilization of nitrate‐based precursors during the initial synthesis phase suggests the existence of N—O bond functionalities in the resulting gels. Typically, these N—O bonds exhibit stretching vibrations within the wavenumber range of 1400–1600 cm^−^^1^ in FTIR spectra.

Furthermore, FTIR analysis reveals the presence of C—N bond functionalities, evidenced by peaks in the approximate wavenumber range of 1500–1600 cm^−^^1^. Additionally, spectra of samples treated at 200 and 400 °C exhibit prominent bands at 1450 and 876 cm^−^^1^, indicative of carbonate groups.^[^ ^28^ ^]^ Crucially, these bands become less intense as the temperature rises. Specifically, following heat treatment at 600 °C, these bands completely disappear from the spectra.

Strong absorption bands in the 400–1000 cm^−^^1^ range are attributed to metal‐oxygen bonds, such as Zn—O, Al—O, and In—O. Additionally, the broadband centered around 965 cm^−^^1^ may include contributions from In‐OH, Zn—OH, and Al—OH bonds. Notably, the distinct band observed at 465 cm^−^^1^ is specifically associated with Zn—O, Al—O, and In—O bonding.^[^ ^29^ ^]^ During thermal treatment, these organic bonds decompose and are eliminated from the structure. FTIR analysis indicates that when ZnAlInO materials are heated to 600 °C, O—H, N—O, and C—N bonds are completely broken down and removed, resulting in the formation of the desired Zn_0.94_Al_0.04_In_0.02_O phase.

### 3.4 Phase Analysis

**Figure 4:**
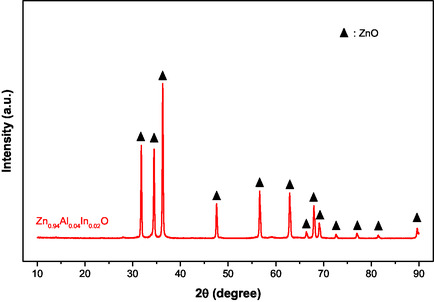
The XRD patterns of Zn_0.96_Al_0.02_In_0.02_O powder materials heat treated at 600 °C for 2 h in air.

**Figure** **4** presents the XRD analysis results for Zn_0.96_Al_0.02_In_0.02_O material synthesized through a 4 h thermal treatment at 600 °C, compared with previously reported data.^[^ ^30^, ^31^ ^]^ The primary diffraction peak corresponds to the ZnO/ZnAlInO phase. Analysis of the 2θ diffraction angles at 31.84°, 34.52°, 36.33°, 47.63°, 56.71°, 62.96°, 68.13°, and 69.18° reveals pronounced preferred orientations, including (100), (002), (101), (102), (110), (103), (200), and (112), characteristic of the hexagonal wurtzite lattice structure of ZnO. The incorporation of small amounts of Al and In into the ZnAlInO lattice is confirmed by these peaks, which match the standard JCPDS card for ZnO (No: 00‐036‐1451). The distinct crystal orientations suggest a high degree of surface alignment, potentially influencing the material's properties.

Compared to literature references,^[^ ^32^, ^33^ ^]^ XRD peaks of Zn_0.96_Al_0.02_In_0.02_O material exhibit slightly broader profiles. This broadening indicates smaller crystal sizes in Al‐ and In‐doped ZnO materials than in pure ZnO materials.^[^ ^32^ ^]^ The incorporation of Al^3^^+^ and In^3^^+^ ions into the ZnO lattice influences its properties due to their distinct chemical characteristics and ionic radii (74 pm). Al^3^^+^ ions, with a smaller ionic radius (53 pm), contract the lattice, while In^3^^+^ ions, larger (80 pm), may expand or subtly distort it. Electrical conductivity is typically improved by doping with Al and In. Al^3^^+^ works as a donor, raising the concentration of carriers in the n‐type semiconductor, while In^3^^+^ ions also introduce more charge carriers, but with different effects.^[^ ^30^, ^31^, ^32^, ^33^, ^34^ ^]^

### 3.5 XPS Analysis

**Figure 5:**
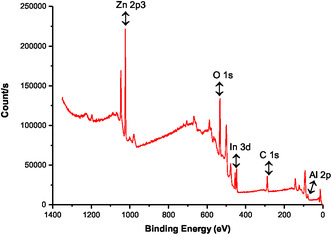
The XPS analysis results of Zn_0.96_Al_0.02_In_0.02_O powder materials.

XPS analysis was carried out to look at the electronic states and surface chemical makeup of ZnAlInO ceramic powder materials. **Figure** **5** illustrates the XPS results for Zn_0.96_Al_0.02_In_0.02_O ceramic powder, detailing binding energies and atomic percentages for Zn 2*p*3, Al 2*p*, In 3*d*, O 1*s*, and C 1*s* regions. **Table** **4** summarizes these findings. This extensive review highlights the possible uses and electrical properties of these cutting‐edge ceramic materials while offering insights into the elemental makeup and bonding environment.

**Table 4:** Elemental identity and quantities of ZnAlInO materials.

Zn_0.96_Al_0.02_In_0.02_O			
Element ID	Binding energy [eV]	FWHM [eV]	Atomic [%]
Al 2*p*	77.17	4.712	15.89
Zn 2*p*3	1022.27	2.935	18.87
In 3*d*	445.87	3.223	2.71
O 1*s*	532.1	3.52	44.07
C 1*s*	286.96	4.02	18.46

Zn is an essential component of thermoelectric materials. One characteristic of the Zn 2*p*3 XPS spectra is that they separate into two doublets, each of which represents a different chemical structure. As reported in reference, the Zn 2*p* core‐level XPS spectra show two strong peaks with binding energies of 1045.00 and 1022.12 eV, respectively, which are attributable to Zn 2*p*_1/2_ and Zn 2*p*_3/2_ of tetrahedral Zn^2+^.^[^ ^35^ ^]^ This validates that the Zn in the ZnAlInO materials is in the +2 oxidation state. Based on the Al and In dopants and their stoichiometric compositions, such as Zn_0.96_Al_0.02_In_0.02_O, the Zn‐binding energy values were found to be 1023.28 and 1022.27 eV, respectively, as indicated in Table 3. The Al 2*p* core peaks in Figure 5 are represented by the two main components, Al 2*p*_3/2_ and Al 2*p*_1/2_, which are situated at 74.74 and 74.14 eV, respectively, for the Al element. Because of the spin–orbit coupling, the Al 2*p* peak is divided into two separate ingredience. Because of density variations, the Al 2*p*_3/2_ ingredience is more noticeable than the Al 2*p*_1/2_ ingredience. The typical oxidation state of Al is around +3, which indicates a greater oxidation state than that seen in sintered materials. This is determined by comparing the relative area of the satellite peaks to the primary Al 2*p* peak. Table 4 shows that the binding energy value of Al, which was affected by the In dopant in Zn_0.96_Al_0.02_In_0.02_O, was 77.17 eV, respectively, as stated in.^[^ ^36^ ^]^

Furthermore, the spectrum of In usually contains peaks that correlate to different core levels, most notably In 3*d*. One of the most often examined peaks for In is the In 3*d* peak, which is seen with a binding energy of 445–452 eV. In order to differentiate between elemental In (In^0^) and oxidized forms like In_2_O_3_ (In^3+^), this peak is essential for determining the oxidation state of In. Depending on the oxidation state, the binding energy of In peaks changes; for example, In^0^ usually shows lower binding energies than oxidized states like In^3+^.^[^ ^37^ ^]^ For In 3*d*, the binding energy for In in Zn_0.96_Al_0.02_In_0.02_O was found to be 445.87 eV.

Critical characteristics necessary for thorough examination are shown by the O 1*s* spectra. Both metal–oxygen bonds (O1 peak) and adsorbed oxygen species (O2 peak) contribute to the O 1*s* peaks, which show clear separation in all samples. These peaks are found to have binding energies of 533.46 and 532.10 eV, respectively. The presence of oxygen vacancies is indicated by the observed shift in the O1 peak, highlighting the significance of analysing oxygen defects in clarifying the material matrix's electrical and structural characteristics.^[^ ^35^, ^36^, ^37^ ^]^

Carbon is usually found in thermoelectric materials as carbon dioxide (CO_2_) and carbon (C) pollutants from the surrounding air. Therefore, it is crucial to carry out material characterisation tests in controlled settings in order to reduce contamination. For the appropriate study and interpretation of the material's structure and qualities, laboratory environments and equipment must be kept clean. The reliability of the data is improved by lowering contamination, which makes it possible to assess the material's performance more precisely. Research utilizing thermoelectric materials must thus pay close attention to cleanliness.

### 3.6 Microstructure

**Figure 6:**
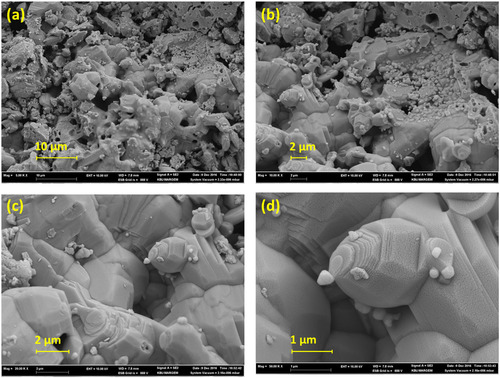
SEM images of Zn_0.96_Al_0.02_In_0.02_O material with different magnifications such as a) 5000x, b) 10000x, c) 20000x, and d) 50000x.

The detailed SEM examination of powder materials provided micrographs at magnifications of 5000x, 10000x, 20000x, and 50000x, shown in **Figure** **6**. The in‐depth investigation of these images confirmed that the grain shapes of the synthesized powders matched the expected geometrical forms, supporting the predicted structural traits. Figure 6a specifically displays the agglomerated structure of Zn_0.96_Al_0.02_In_0.02_O powder at 5000x magnification. The powders consist of tiny solid particles with a strong tendency to clump together. Agglomeration involves individual powder particles merging into larger clusters due to forces such as gravity, collisions, electrostatic effects, coagulation and filtration, and chemical interactions. The behavior of these processes depends significantly on surrounding environmental circumstances and the medium containing the powder.^[^ ^38^ ^]^

Within the ZnAlInO structure, hexagonal configurations form the prevailing, globally regular morphologies. However, these hexagonal forms consist of very tiny particles that have clustered into greater masses. The size of these aggregated clusters spans 0.3–2.0 μm, with each particle measuring between 25 and 150 nm. Figure 6b,c provide a closer look at the microstructures of the agglomerated powder shown in Figure 6a, magnified to 10000x and 20000x, respectively.

Figure 6d micrographs show the detailed regular‐shaped particle morphology. These observations align with previously reported findings,^[^ ^39^ ^]^ briefly outlined here. Particles with regular shapes, featuring varying corners and thicknesses up to 500 nm, are observed. These particles’ thicknesses differ greatly, from around 200 to 500 nm, with larger grains showing a particularly typical morphology. Conversely, Figure 6d highlights the presence of notable layered structures within the ZnAlInO composition. These layers have a thickness ranging from 20 to 25 nm.

Complete SEM examination confirms the expected forms and clustering behavior while validating the synthesized powders’ morphological homogeneity and structural integrity. This study not only corroborates previous research results but also lays a solid groundwork for future research and industrial applications involving these materials.

### 3.7 Thermoelectric Results

**Figure 7:**
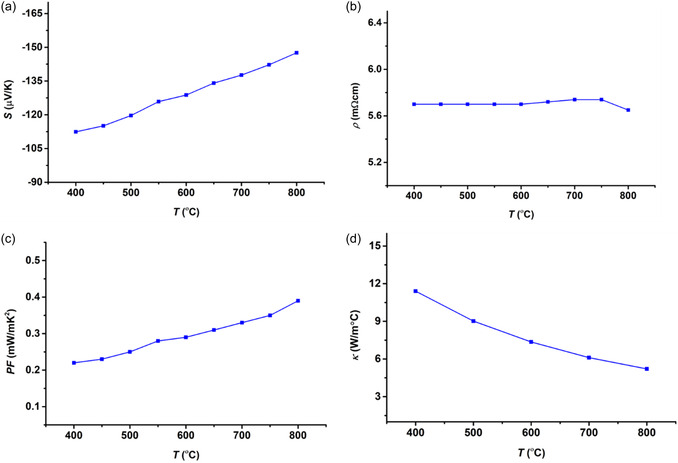
Thermoelectric properties of Zn_0.96_Al_0.02_In_0.02_O material with respect to temperature: a) Seebeck coefficient, b) electrical resistivity, c) power factor, and d) thermal conductivity.

The ceramic modules were produced using the CP process with ceramic powder, allowing for an analysis of the thermoelectric properties of Zn_0.96_Al_0.02_In_0.02_O bulk material. The results are detailed in **Figure** **7**. Thermal conductivity was measured every 100 °C, while electrical resistivity and Seebeck coefficients were measured at 50 °C intervals across the temperature range of 400–800 °C. The conduction mechanism of the n‐type semiconductor was confirmed by negative Seebeck coefficients, consistent with previous research.^[^ ^13^ ^]^ With the addition of example templates, Seebeck coefficients increased, reaching a maximum of −162.63 μV K^−1^ at 800 °C, highlighting the advantages of concurrent interactions in textured ceramics. These interactions enhance Seebeck values by reducing low‐energy carrier scattering. The power factor values, also shown in Figure 7, improved with template inclusion, reaching a peak of 0.44 mW m^−1^ K^−2^ at 800 °C. The thermal conductivity of the samples was found to be 3.96 W m^−1^ K within the tested temperature range. Comparing the obtained Seebeck coefficient, power factor, and thermal conductivity values with the literature, it is clear that Zn_0.96_Al_0.02_In_0.02_O exhibits significantly better properties. This comparison is shown in **Table** **5** and referenced in the literature.^[^ ^40^, ^41^, ^42^, ^43^, ^44^ ^]^ Consequently, the obtained Seebeck coefficient, power factor, and thermal conductivity values allow for a detailed comparison.

**Table 5:** Summary comparison of thermoelectric materials.

Material	Seebeck coefficient [μV K^−1^]	Power Factor [mW m^−1^ K^−2^]	Thermal conductivity [W m^−1^ K^−1^]	Reference
ZnO (doped)	−20–−50	0.05–0.3	5.0–7.0	[40]
ZnAlO	−20–−60	0.1–0.3	4.0–6.0	[41]
ZnAlGeO	−30–−60	0.05–0.2	4.5–6.5	[42]
ZnAlGaO	−25–−70	0.05–0.2	4.0–5.5	[43]
ZnAlInO	−50–−90	0.2–0.5	3.5–4.5	[44]

The Seebeck coefficient for Zn_0.96_Al_0.02_In_0.02_O reached −162.63 μV K^−1^ at 800 °C, which is significantly more negative than the range reported for other materials (Table 5). This suggests enhanced Seebeck values due to template inclusion and interactions in textured ceramics, which help reduce low‐energy carrier scattering. The power factor for Zn_0.96_Al_0.02_In_0.02_O reached 0.44 mW m^−1^ K^−2^ at 800 °C, aligning with the higher end of the values listed in the table. This indicates that template inclusion improves the power factor, enhancing thermoelectric efficiency. The thermal conductivity for Zn_0.96_Al_0.02_In_0.02_O was measured to be 3.96 W m^−1^ K^−2^ within the temperature range of 400–800 °C, which is relatively lower compared to other materials in Table 5. This suggests that it may be better suited for thermoelectric applications, as lower thermal conductivity enhances thermoelectric device efficiency. As a result, this shows that ZnAlInO samples are suitable for potential aerospace applications.

## 4 Conclusion

This study successfully achieved the synthesis and production of n‐type ZnAlInO semiconductor materials for TEG fabrication for aerospace implementation, utilizing the CP and sol–gel methods. The outcomes derived from experiments can be found in the following list: a) Experimental findings indicate turbidity values of 0.52 ntu for Zn_0.96_Al_0.02_In_0.02_O solutions, respectively, indicating the extent of precursor powder dissolution in these solutions. The pH range of 1.26 in the prepared solutions reflects an acidic environment, which facilitates gel formation; b) based on DTA‐TG analysis results, the heat treatment protocol for ZnAlInO involved preliminary drying at 200 °C for 10 h, followed by oxidation and calcination at 600 °C for 4 h; c) According to FTIR analysis, heating ZnAlInO powders to 600 °C fully removes O—H, C—N, and N—O bonds, creating the desired phases in the material; d) small quantities of Al and In were added to Zn_0.96_Al_0.02_In_0.02_O to create the hexagonal wurtzite lattice structure, which was validated by the XRD patterns; e) XPS examination detected Al and In dopants in Zn_0.96_Al_0.02_In_0.02_O powder, confirming the effective fabrication of n‐type semiconductor materials; f) detailed SEM analysis verified the anticipated geometric forms and agglomeration behavior, verifying the produced powder materials’ morphological uniformity and structural integrity; g) Seebeck coefficient increased with the appendance of template samples, reaching a maximum of −162.63 μV K^−1^ at 800 °C for Zn_0.96_Al_0.02_In_0.02_O. Power factor values rose with template additions, achieving a peak of 0.44 mW mK^−2^ at 800 °C. The thermal conductance of the Zn_0.96_Al_0.02_In_0.02_O sample was determined to be 3.96 W mK^−1^ inside the temperature range that was tested.

## Conflict of Interest

The authors declare no conflict of interest.

## Author Contributions

**Enes Kilinc**: conceptualization (equal); formal analysis (equal); investigation (lead); methodology (equal); validation (lead); visualization (equal); writing—original draft: supporting; and writing—review and editing: supporting. **Fatih Uysal**: formal analysis (equal); investigation (equal); validation (equal); and writing—review and editing: supporting. **Mucahit Abdullah Sari**: investigation (equal); visualization (lead). **Huseyin Kurt**: conceptualization (equal); funding acquisition (lead); project administration (lead); resources (lead); and supervision (lead). **Erdal Celik**: conceptualization (lead); formal analysis (lead); methodology (lead); resources (equal); supervision (equal); writing—original draft (lead); and writing—review and editing (lead).

## Data Availability

The data that support the findings of this study are available from the corresponding author upon reasonable request.
